# The H-index is an unreliable research metric for evaluating the publication impact of experimental scientists

**DOI:** 10.3389/frma.2024.1385080

**Published:** 2024-07-19

**Authors:** M. Kalim Akhtar

**Affiliations:** Department of Chemistry, College of Science, United Arab Emirates University, Al Ain, United Arab Emirates

**Keywords:** original research, perspectives, reviews, citations, scientific reputation, research community, metric abuse

## Introduction

The H-index is a widely used research metric for assessing the reputation of scientists. It is a numerical indicator that measures publication impact (Hirsch, 2005). The value is determined by taking the “*h*” number of publications that have been cited at least “*h*” times. The higher the *h*-index the greater the publication impact of the scientist. Table 1 shows the publication profile of 6 scientists who are all academically engaged within the experiment-driven research areas of the biomolecular sciences. The publication and citation statistics for these scientists were obtained from the Scopus database during April 2023. Profiles 1–5 belong to five renowned prize-winning scientists who have been awarded the Nobel Prize in either the “Chemistry” or “Physiology and Medicine” category and/or the “Breakthrough Prize in Life Sciences” for ground-breaking experimental research (refer to Table 1 for list of names). Both prizes are prestigious and awarded for ground-breaking experimental work. The awardees of these prizes are Robert S. Langer (prolific inventor in biomedical engineering), Michael Houghton (trailblazer in vaccine development), Katalin Karikó (pioneer in RNA therapeutics), Jennifer A. Doudna (pioneer of CRISPR technology), and Shankar Balasubramanian (innovator of DNA sequencing). Their H-indexes vary from 51 to 237. For brevity, I will collectively refer to them as pre-eminent scientists. The final scientist on this list, who I shall refer to as Scientist X, is also a biomolecular scientist and has an H-index of 64. Unlike the pre-eminent scientists, Scientist X is not internationally recognized nor been awarded any major science prizes. Curiously, Scientist X features in the Clarivate database of highly cited researchers. Moreover, this scientist's average citations per year exceeds the average citations of the pre-eminent scientists (except for two). How is it conceivable that a scientist with no distinguished track record in an experimental field can generate more citations than prize-winning scientists? The answer to this question, as I shall reveal here, is due to a meretricious publication output rather than experimental novelties or innovations of any kind.

**Table 1:** H-index profiles and publication statistics of 6 highly cited scientists in 2022.

**Profile**	PY^*^	**Majorawards**	**Area of expertise**	**H-index^+^**	**H5-index^+^ (2018–2022)**	**Average citations^++^ (per publishing year)**	**Citations (2018–2022)**	ORA^**^ (2018–2022)	RPA^***^ (2018–2022)	**Ratio of ORA:RPA (2018–2022)**	**% of citations from RPA contributing to the H5-index (2018–2022)**	**EH-index^+#^**	**EH5-index^+#^ (2018–2022)**
Robert S Langer	48	Breakthrough Prize in Life Sciences, 2014	Tissue engineering	237	47	5,425	12,698	129	30	4:1	30%	203	37
Michael Houghton	48	Nobel Prize, 2020	Vaccine development	88	11	904	326	16	3	5:1	28%	81	10
Katalin Karikó	33	Breakthrough Prize in Life Sciences, 2022 Nobel Prize, 2023	RNA therapeutics	51	17	511	2,811	17	3	5:1	4%	45	13
Jennifer A Doudna	35	Nobel Prize, 2020; Breakthrough Prize in Life Sciences, 2015	Genome editing	114	36	1,975	7,683	72	11	7:1	1%	102	34
Shankar Balasubramanian	32	Breakthrough Prize in Life Sciences, 2022	DNA sequencing	85	26	1,211	3,125	40	4	10:1	26%	82	24
Scientist X	12	None	Molecular science	64	55	1,629	12,921	120	324	1:2	87%	34	24

^*^PY, Publishing years; ^**^ORA, Original Research Articles; ^***^RPA, Review and Perspective Articles. ^+^Self-citations are excluded. ^++^This value was calculated by taking total publication citations and dividing by the number of publishing years. ^#^Newly proposed index based on the impact coming from experimental articles and excludes impact from non-experimental articles such as reviews and perspectives.

## Prize-winning scientists build their scientific reputation primarily on experimental work

To fairly evaluate the publication impact of these scientists whose careers span different decades, we can employ the H5-index to quantify their publication impact over the last 5 years from 2018 to 2022. The H5-indexes of the prize-winning scientists range from 17 to 47. Scientist X, however, trumps them all with an H5-index of 55. From a publication viewpoint, one might conclude that Scientist X is the more impactful scientist. But where is this impact coming from?

Scientists regularly communicate their ideas and findings via original research articles and secondary source articles such as reviews and perspectives. An original research article embodies an experimental study that leads to new data and findings. A review or perspective article describes experimental progress or presents new viewpoints within a particular research field and relies on data from original research articles. Table 1 summarizes these two main types of publications for all 6 scientists over the 5-year period from 2018 to 2022. During this period Scientist X published in total 324 review and perspective articles. This figure is almost the same as the total number of publications of all the prize-winning scientists put together, in this case 330. This astonishing statistic is a clear example of overpublishing, an issue that is now prevalent in science publishing as highlighted by Akbashev and Kalinin (2023). Typically, a scientist would write a review or perspective after they have made significant experimental contributions. The prize-winning scientists, on average, write one review/perspective paper for every six original research articles. Scientist X, on the other hand, writes two review/perspective papers per original research article. Clearly then, Scientist X gives an inordinate amount of attention to literature-based work rather than original research work to generate publication impact.

## Non-experimental work provides a fast-track route for increasing the H-index

But why is Scientist X giving so much attention to reviews and perspectives rather than experimental studies? For the simple reason that reviews and perspectives offer a faster and easier track to a high H-index value. Review and perspective articles can be completed within a few weeks in contrast to months or years for experimental work. Provided the authors are well versed in their fields and choose a subject matter that is relevant and aligned to current research trends, the writing of a review or perspective article is a straightforward process. A perspective article relating to marine plastic waste that was published recently took me only 3 weeks to write from the conception of the idea to the final submitted version and that was without the assistance of text generators or literature analysis software (Alnahdi et al., 2023). With AI-based tools, this entire writing process could be expedited and-upscaled with powerful qualitative data analysis software and text-generating applications designed to survey the literature and produce textual material. Experimental work, on the other hand, is a much slower affair due to the requirement for technical expertise, access to scientific instrumentation, along with the unpredictable nature of experiments. For instance, a recent experimental study carried out by my team in collaboration with two other research teams took almost 2 years to complete from conception of the idea(s) to the final revised article (Baby et al., 2023). To make matters worse, the time frame can be prolonged or even delayed if research teams are poorly funded and work with inadequate resources. In such unfortunate circumstances, relentless pursuit of experimental work will most certainly not lead to a high H-index value and may even be detrimental to the scientist's career. Most scientists would agree that a study of an experimental nature is a far more difficult and time-consuming ordeal compared to writing a short perspective or a review article.

## Issues concerning the overpublication of non-experimental articles

From an experimental standpoint, the publishing behavior of Scientist X is concerning for several reasons. Firstly, H-index values generated in this way creates the false and misleading impression that such scientists have amassed a large and credible body of experimental work within their field of specialty. Worse still, it sets them on the same pedestal with far more accomplished experimental scientists with similar H-indexes. Secondly, this publishing behavior highlights a major shortcoming of the H-index, namely that the metric does not allow one to quantify the scientific impact coming solely from field or laboratory-based work. By giving equal weighting and importance to other types of publications such as reviews and perspectives which are far easier to publish, the H-index value becomes prone to hyperinflation. Thirdly, due to its simplicity and effectiveness in boosting the H-index, this overpublishing strategy could easily be replicated by scientists looking to hyperinflate their H-index values. Finally, and quite worryingly, this may well encourage a future generation of scientists to divert their attention away from experimental work.

## Quantitating publication impact from experimental work

To counteract behavior associated with the overpublication of reviews and perspectives, the H-index could be further refined so that the publication impact of scientists is quantified from their experimental work. This could be referred to as the EH-index where E is an abbreviation for “experimental.” Similarly, the EH-5 index would be the scientific impact accrued over a period of 5 years. By excluding review papers as well as other types of secondary source articles, the EH-index would more accurately reflect the impact coming from the experimental work of active scientists within their field of specialties. Publication impact attributed to other types of articles such as reviews, perspectives, opinions, and even meta-analysis papers could be quantified under separate publication categories.

It should be noted out that what is being proposed here is not a new metric but a subtle modification of an existing one. The EH-index would specifically quantitate the publication impact from experimental studies. Given that science articles are categorized into various article types such as review, editorial, book chapter, etc, it should be possible to automate the process for determining the EH-index. Unfortunately, at the database level (in the case of Scopus and Web of Science databases), inaccuracies still exist when it comes to filtering out original research articles. This would need to be corrected before the EH-index could be made available.

Research metrics quite often rely on total citation counts which are weighted using a specific criterion. For example, the field-weighted citation impact (FWCI) is the total citation count in relation to the average citation impact for a particular research field. Interestingly, a research metric that weights total citation counts according to the type of research contribution does not exist. Thus, the EH-index proposed would be the first research metric of its kind based on citations coming solely from experimental work.

Of course, this approach is not limited to the H-index and could be applied to other author metrics such as the G-index and M-index. Given the overcitation and overrepresentation of review papers (Miranda and Garcia-Carpintero, 2018), one could even extend this concept to journal metrics such as the impact factor so that it becomes easier to identify journals that have built their reputations on publishing experimental work. Using this newly proposed EH-index, the data relating to the publication and citations of all 6 scientists is presented in Table 1. The EH5-indexes, which quantifies their output over the last 5 years from 2018 to 2022, is also given in this table. Notice that the EH-index value of Scientist X who relies mostly on secondary source articles, such as reviews and perspectives, for creating impact is substantially lower than the H-index, by as much as 56%. For the prize-winning scientists, this drop varies from 1 to 24%. Curiously, this suggests that prize-winning scientists may also succumb to this type of behavior. I would speculate here that such scientists are more than likely invited on a regular basis by editors of top-tier journals to contribute state-of-the-art reviews and perspectives, and view these journal invitations as excellent public relations opportunities for promoting their research discipline to a large science audience.

## Conclusion

After more than 10 years of implementing the H-index as a quantitative metric, academics have figured out how to game this metric in a number of ways, as described recently by Oransky et al. (2023). Koltun and Hafner (2021), after analyzing the H-indexes of millions of researchers, reached the conclusion that there is no longer any correlation between the H-index and scientific reputation. This disconnect between the H-index and scientific reputation I believe is the result of a science culture that is fast relying on disingenuous strategies and approaches to improve publication statistics, a view supported by Chapman et al. (2019).

A major shortcoming of the H-index is its failure to distinguish between an original research study and a literature-based one. Underlying the H-index metric is the incorrect assumption that the time, effort and even funding required for an original research study is the same as that for other publication types. In this regard, the H-index is an abysmal metric for evaluating experimental researchers (Bi, 2023). Original research work is the principal driving force for scientific progress, not reviews and perspectives. Thus, an alternative experiment-oriented metric such as the EH-index proposed here is sorely needed to ensure a higher standard of publishing in scientific research. This newly proposed index would be of great benefit to academic and research institutes as it would allow institutes to identify scientists that can create impact, via original experimental work. Research output from such individuals will be absolutely crucial for developing innovative scientific models and applications.

Given our complete dependence on publication metrics for evaluative purposes, it is worth questioning the behavioral trends that have emerged amongst scientists to game these metrics. Is there a genuine field-wide correlation between publication of reviews, as well as perspectives, and the inflation of H-index values? What percentage of scientists might be following this practice of overpublishing secondary source articles to boost their H-index? Are other research metrics besides the H-index also impacted by these publishing trends? Does it extend to other experimental disciplines including those within the social sciences? Should we be overly concerned by these strategies to over-inflate research metrics? Do they create a better standard for publishing? If not, what can we do to counteract this and instead encourage behavior that will improve the research culture? These are all questions that merit further investigation.

It stands to reason that any type of metric once it is gamed will eventually be subject to abuse (Bi, 2023). Although the proposed EH index would not completely stop malpractice or unethical behavior to boost one's research status, it will at least increase transparency of a scientist's publication output and shift the focus away from reviews and perspectives which consumes a lot of review time and detracts from the importance of experimental innovations. Regardless of the quantitative metric employed and in accordance with the Leiden Manifesto (Hicks et al., 2015), qualitative factors (*e.g*. journal prestige, reputation of collaborators, membership of editorial boards) should not be neglected when assessing the reputation and productivity of scientists.

## Author contributions

MA: Conceptualization, Data curation, Formal analysis, Funding acquisition, Investigation, Methodology, Resources, Visualization, Writing – original draft, Writing – review & editing.
